# 

**Published:** 1906-03-15

**Authors:** 


					﻿CONTENTS.
Event and Comment -	-	-	-	~	~	-109
Compressed Air, - -Mt .ril - By Ge<k Zederbaum, D.D.S. 115
High Pressure Anesthesia,	-	-	-	M. Weaver,	D.D.S. 128
Obtundingr Dentin,	—--——... By Ellison Hillyer,	D.D.S. 134
With Our Editors	-	-	-	-	-	-	- -	-	-141
Indents -----------	14g
Announcements	-	-	-	-	-	-	- -	-	-158
				

